# Synthesis and Characterization of Bio-Oil Phenol Formaldehyde Resin Used to Fabricate Phenolic Based Materials

**DOI:** 10.3390/ma10060668

**Published:** 2017-06-18

**Authors:** Yong Cui, Xiaopeng Hou, Wenliang Wang, Jianmin Chang

**Affiliations:** 1 Precision Manufacturing Engineering Department, Suzhou Vocational Institute of Industrial Technology, Suzhou 215104, China; 00767@siit.edu.cn; 2 Research Institute of Wood Industry, Chinese Academy of Forestry, Beijing 100091, China; hello168429@sina.com; 3 College of Materials Science and Technology, Beijing Forestry University, Beijing 100083, China; wangwenliang@sust.edu.cn; 4 College of Bioresources Chemical and Materials Engineering, Shaanxi University of Science & Technology, Xian 710021, China

**Keywords:** bio-oil, phenol formaldehyde resin, paraformaldehyde, synthesis and characterization

## Abstract

In this study, bio-oil from the fast pyrolysis of renewable biomass was used as the raw material to synthesize bio-oil phenol formaldehyde (BPF) resin—a desirable resin for fabricating phenolic-based material. During the synthesis process, paraformaldehyde was used to achieve the requirement of high solid content and low viscosity. The properties of BPF resins were tested. Results indicated that BPF resin with the bio-oil addition of 20% had good performance on oxygen index and bending strength, indicating that adding bio-oil could modify the fire resistance and brittleness of PF resin. The thermal curing behavior and heat resistance of BPF resins were investigated by differential scanning calorimetry (DSC) and thermal gravimetric analysis (TGA). Results showed that adding bio-oil had an impact on curing characteristics and thermal degradation process of PF resin, but the influence was insignificant when the addition was relatively low. The chemical structure and surface characteristics of BPF resins were determined by Fourier transform infrared (FTIR) spectroscopy and scanning electron microscopy (SEM). The analysis demonstrated that adding bio-oil in the amount of 20% was able to improve the crosslinking degree and form more hydrocarbon chains in PF resin.

## 1. Introduction

Through a long history of development, phenol formaldehyde (PF) resin-based materials have been widely used in many fields, thanks to their desirable properties of high rigidity, good corrosion resistance, and less smoke and toxicity when burning. However, the environmental stress and price fluctuation caused by petroleum-based phenol obtained from fossil resources has severely limited the application of PF resin-based material. Therefore, industry efforts have been focused on identifying phenolics from renewable resources.

Biomass is an important renewable source for energy and chemical feedstock, considered as an effective alternative to fossil resources [1,2,3]. Pyrolysis is a thermochemical conversion technology and plays an important role in the utilization of biomass resource. Relevant studies have demonstrated that bio-oil derived from fast pyrolysis of biomass contained a large phenolic fraction and was well suited for creating chemicals [4,5,6,7,8,9]. There were ambitious efforts to substitute phenol in PF resins by phenolic fraction from bio-oil and the synthesis of bio-oil phenol formaldehyde (BPF) resin could be promising in this context. Chan et al. (2002) [10] replaced 25 wt % and 35 wt % of phenol in PF with phenolic-rich oil derived from softwood bark residues vacuum pyrolysis and developed the new type of BPF resin for wood adhesive. It was concluded with the performance of BPF resin that bio-oil can replace up 35 wt % in PF resin for OSB (Oriented Strand Board) manufacturing. Aslan et al. (2015) [11] found that the phenol-rich fraction of crude bio-oil could partially substitute the petroleum-based phenol in commercial PF resin adhesives, and there was a similar molecular structure between BPF and PF resin. Lee et al. (2014) [12] showed that bio-oil blended with phenol was a suitable raw material for preparing alcohol-soluble PF resin, which possessed similar characteristics of thermal melting and setting. Amen-Chen et al. (2002, 1,2,3.) [13,14,15] synthesized BPF resin with 50 wt % of phenol replacement by phenolic-rich bio-oil, and the resin was suited for OSB adhesive. Czernik and Bridgwater [16] showed that 30–50 wt % of phenol could be replaced by bio-oil in the synthesis of PF resin, which were used as adhesives in the manufacture of plywood and particle board. 

Based on previous research, utilizing bio-oil or its phenolic fraction as a phenol source to prepare resol PF resin was feasible, and the BPF resins created with lower cost and toxicity had been proved to be an effective wood adhesive. However, BPF resins used for adhesives were unable to meet the requirement when fabricating resin-based material because of low solid content, high viscosity, and high curing temperature. A distillation processes was needed to improve the properties of traditional BPF resins, yet that would cause increased costs and waste water discharge. In addition, due to the relatively low activity of bio-oil, the adhesive properties of BPF resin synthesized with phenol replacement by bio-oil was weaker than that of commercial PF resin.

In this study, solid paraformaldehyde was used to replace formaldehyde solution (37 wt %) during BPF resins synthesis, aiming to get the properties of high solid content and low viscosity without the distillation processes. In order to maintain the inherent structure of PF resin, the content of synthetic phenol from the petrochemical industry and formaldehyde remained consistent when adding bio-oil to prepare BPF resins. The performance of BPF resins was tested using corresponding standards. The characteristics of BPF resins in terms of curing behavior, curing kinetics, thermal gravimetric properties, chemical structure, and surface characteristics will be described. 

## 2. Materials and Methods

### 2.1. Materials

Dried larch (*Larix gmelinii*) sawdust with dimensions <1 mm was pyrolysised by a fluidized bed at 500–510 °C, which belonged to the Institute of Wood Based Material, Beijing Forestry University, Beijing, China. The liquid products were subsequently extracted with ethyl alcohol (Ke Mi Chemical Company, Tianjin, China), washed by distilled water, and neutralized with sodium hydrogen carbonate (Ke Mi Chemical Co., Ltd., Tianjin, China). The obtained bio-oil contained ingredients of 22% phenolic, 24% ketone, 20% aldehydes, 16% organic acids, 8% sugars, 4% hydrocarbons and other compounds, as determined by GC-MS analysis. The solid paraformaldehyde and phenol (Ruisheng Co. Ltd., Linyi, China) were reagent-grade and used as obtained without purification.

### 2.2. Synthesis of BPF Resins

The synthesis of BPF resins was achieved by using bio-oil, with addition rates of 0 wt %, 10 wt %, 20 wt %, 30 wt %, and 40 wt %. related to the mass of synthetic phenol from the petrochemical industry. The mole ratio of synthetic phenol to formaldehyde was consistently maintained as 1:1.8 in the resin, regardless of how much bio-oil was added. The BPF resins prepared with various amounts of bio-oil were designated as PF, 10%-BPF, 20%-BPF, 30%-BPF, and 40%-BPF, respectively. The mole ratio of NaOH to synthetic phenol was always set as 0.25:1.

The phenol and 70% NaOH solution (30 wt %) were mixed in a 250-mL three-neck round-bottom glass reactor equipped with a stirrer, thermometer, and reflux condenser. The reactant was heated to 70 °C in 30 min. Then, 80% paraformaldehyde was gradually added and the temperature was held at 70–75 °C under continuous stirring until completely depolymerized. Next, the remaining 30% of NaOH solution (30 wt %) was added, and the reactant was heated to 80 °C in 30 min and kept for 15 min. After the temperature was dropped to 70 °C, the remaining 20% of paraformaldehyde and bio-oil was gradually added. The reactant was heated to 85–90 °C and held for 45 min. When the reaction was complete, it was rapidly cooled to 40 °C to yield the BPF resins.

### 2.3. Preparation of Resin Casting Model

The BPF resin castings with the size of 150 × 6.5 × 3 mm³ (length × width × thickness) were separately prepared to meet the tests of bending strength and oxygen index, using acidic curing agent at room temperature.

After surface clearing and drying, the mold was coated with solid paraffin as the release agent. For each resin casting, acidic curing agent (0.35 g) was gradually added to resins (7 g) and the mixture was blended. However, to make it easy to prepare, the amount of resins that was blended for one time should be used in six or eight castings. Next, the resin mixture was poured into the mold and laid for 24 h at 25 °C to cure. After demolding and further curing at 70 °C for 2 h, the casting model was finally prepared.

### 2.4. Characterization of BPF Resins

The viscosity, solid content, free formaldehyde, and free phenol of BPF resins were determined in accordance with the Chinese National Standard (GB/T 14704-2006). The bending strength and oxygen index of BPF resins were measured according to Chinese National Standard (GB/T 1449-2005, GB/T 8924-2005). Oxygen index was defined as the minimum volume fraction of oxygen in the mixed gas in which the resin casting could be fired. Oxygen index was used to investigate the property of fire resistance, and was measured by special equipment with the resin coasting prepared as the size of 150 × 6.5 × 3 mm³ (length × width × thickness).

The thermal curing behavior was analyzed by a differential scanning calorimeter (DSC; Perkin-Elmer, Waltham, MA, USA) in a nitrogen atmosphere. Uncured resins were heated from 40 °C to 200 °C at heating rates of 5, 10, 15, and 20 K·min^−1^ in aluminum pans. The thermal degradation processing of uncured resins was conducted by thermal gravimetric analysis (TGA) (Perkin-Elmer, Pyris 1, Waltham, MA, USA) in a nitrogen atmosphere. The scanning temperature ranged from 50 to 850 °C with the rate of 10 °C·min^−1^. The flow rate of nitrogen in DSC and TGA analysis was 20 mL·min^−1^. The functional groups of uncured and cured resins were characterized by FTIR (Bruker Vertex 70, Karlsruhe, Baden Wurttemberg, Germany). The Fourier transform infrared (FTIR) spectra of BPF resins ranged from 4000 to 400 cm^−1^. The surface characteristics of cured resins were observed by SEM (Quanta-200, FEI Company, Hillsboro, OR, USA) at an accelerating voltage of 5000 V. The uncured resins were freeze-dried before DSC, TG, and FTIR analysis. The cured resins were prepared by treating uncured resins at 120 °C for 2 h in an air convection oven.

## 3. Result and Discussion 

### 3.1. Performance of BPF Resins

As shown in Table 1, the BPF resins had lower viscosity and solid content than PF resin, which was becoming more obvious with increasing bio-oil addition. It is considered that the resin could be diluted by bio-oil containing water and other organic compounds. The viscosity of resins should meet the requirement of fabricating phenolic-based materials, and the lower viscosity was beneficial for operation. The results showed that adding bio-oil was able to reduce the viscosity. The BPF resins synthesized by paraformaldehyde without water distilling had solid content over 70%. 

As the bio-oil addition increased, the free formaldehyde content of BPF resin was gradually reduced, but the free phenol content continuously enhanced. This showed that natural polyphenols in bio-oil can undergo hydroxyl-methylated reaction with formaldehyde as the synthesis process, and most of the formaldehyde was consumed, but excessive natural polyphenol would raise the content of free phenol. The low-toxicity resin needed the free formaldehyde and phenol to be below the limited requirement at the same time.

Oxygen index and bending strength of resin casting model reached their maximum values at the bio-oil addition of 20%, which proved that bio-oil can improve the brittleness and fire resistance of PF resin. Bio-oil includes plenty of active ingredients, such as phenols, aldehydes, ketones, and hydrocarbon, and can react with a PF resin system, the performance of which is thus improved. However, adding too much bio-oil to the formaldehyde–phenol system would interfere in the formation of the inherent crosslinking structure and have a negative effect on the performance of the resin.

The results indicated that BPF resin synthesized by suitable proportions of bio-oil and paraformaldehyde exhibited good performances compared to prepared resin-based materials.

### 3.2. DSC Analysis

**Figure 1:**
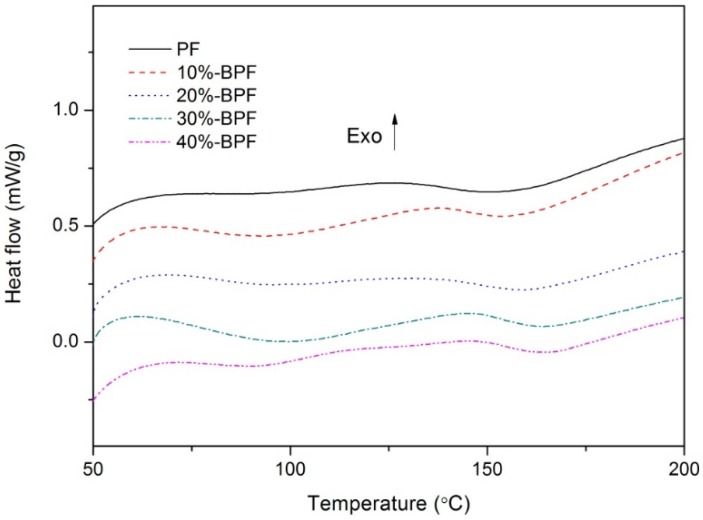
Differential scanning calorimetry (DSC) curves of various BPF resins (10 °C·min^−1^).

The DSC curves of the various BPF resins at the heating rate of 10 °C·min^−1^ are displayed in Figure 1. The peak temperatures obtained at different heating rates and their corresponding calculation results are listed in Table 2. 

The surfaces of exothermic peaks of BPF resins were larger than that of PF resin, and the peak temperature values increased with the increased bio-oil addition, indicating that adding bio-oil may reduce the reactivity of PF resin. The results of curing temperature—determined from linear-regression analysis of peak temperatures at different heating rates [17,18]—also showed that higher temperature was needed to make resins cured in the case of increasing bio-oil addition. However, as the bio-oil addition was below 20%, the curing temperature of BPF resins was quite close to that of PF resin.

The activation energy was obtained by the Flynn–Wall–Ozawa or Kissinger method, depending on the peak temperature at different heating rates [19]. As shown in Table 2, the activation energy values calculated by the two methods were similar, although the values produced by the Flynn–Wall–Ozawa method (E_f_) were always slightly lower than the values produced by Kissinger method (E_k_). All the regression coefficient values were more than 0.98, proving that the results calculated by the above methods were credible. The activation energy values of BPF resins were higher than that of PF resin, and that phenomenon became more obvious with the increasing amount of bio-oil addition. It was also demonstrated that the BPF resins with relatively high bio-oil addition (e.g., 30%-BPF and 40%-BPF) required much more energy to cure completely than those having low bio-oil addition.

As mentioned above, the differences of the curing properties between PF and BPF resins were mainly attributed to the lower chemical activity of bio-oil, which may cause serious steric hindrance during the curing process. However, as compared to the data of curing temperature and activation energy in Table 2, the BPF resins with bio-oil addition of 10–20% had similar curing characteristics as PF resin. 

### 3.3. TGA Analysis

**Figure 2:**
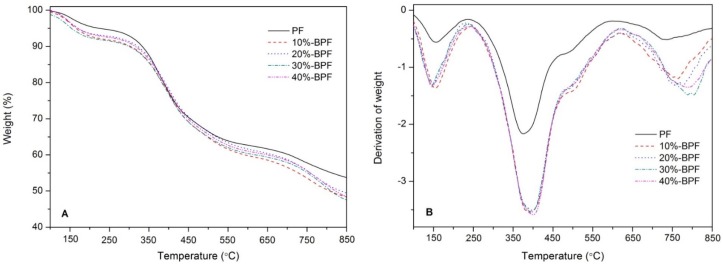
(**A**) Thermal gravimetric analysis (TGA) and (**B**) derivative TGA (DTG) curves of various BPF resins.

The TGA and derivative TGA (DTG) curves are summarized in Figure 2A,B. The results indicated that the thermal degradation of BPF resins was similar to that of PF resin during the temperature range of 50–850 °C, divided into three regions: I, <300 °C; II, between 300 and 650 °C; III, >650 °C. In region I, the weight loss was due to the evaporation of water and small molecular substances. The evolution of water derived from the physical desorption or the condensation reaction between hydroxymethyl groups. Most of the small molecular substances (e.g., free formaldehyde, free phenol, and cresol) were released at a higher temperature [20]. In region II, a major weight loss took place. The formaldehyde was from the cleavage of ether bonds formed in curing solution or dehydrogenation of the methylol groups from the aromatic rings [21]. The methylene radical that was scissored out of the polymer chain can react with hydrogen to form CH_4_. Some phenolic hydroxyls were stripped off, and hydroxyl radicals were generated correspondingly. The methylene and hydroxymethyl could be oxidized by hydroxyl radicals to create the structures of carbonyl and carboxyl [22], of which the release of CO and CO_2_ may come from the further reactions. A few of benzene and its derivatives came out of the methylene bridge and ether bridge breaking. Besides, reactions between phenolic hydroxyl and methylene or two hydroxyl groups resulted in the release of water. In region III, the weight loss was mainly caused by the dehydrogenation of benzene rings [23].

As clearly shown, the temperature-dependent weight loss of BPF resins were more than in the case of PF resin, indicating that the carbohydrates and carbides of bio-oil were easily volatilized at higher temperature. However, as compared the TGA and DTG curves of various resins, the gap between the maximum weight loss of 54.6% and minimum 49.7%—attributed to 40%-BPF and PF resin, respectively—actually appeared to be slight. This demonstrated that adding bio-oil had a limited impact on degrading the heat resistance.

### 3.4. FTIR Analysis

**Figure 3:**
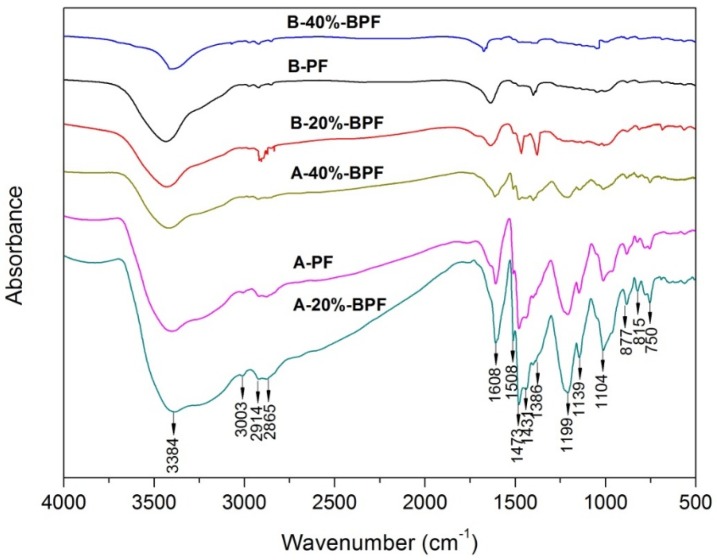
Fourier transform infrared (FTIR) spectrum of various uncured (**A**) and cured (**B**) BPF resins.

The uncured and cured BPF resins were analyzed using the FTIR spectrum in Figure 3, to explore the chemical structure of resins produced. It used to be clear that the peak assignments of FTIR spectrum of BPF resins were similar to that of PF resin. However, there were distinct differences among BPF resins in the intensity of peaks belonging to feature functional groups, due to the addition of various amounts of bio-oil. 

Figure 3A shows the FTIR spectrum of the uncured BPF and PF resins. The broad peak at 3384 cm^−1^ was assigned to the stretching vibration of hydroxyl [24], and the stretching vibration of phenol C–O at 1199 cm^−1^ and benzyl hydroxyl C–O at 1104 cm^−1^ [25] suggested that there were residual phenolic hydroxyl and hydroxyl methyl. The benzene or its derivatives were concluded by the stretching vibration of benzene C–H at 3003 cm^−1^ and benzene C=C at 1608 cm^−1^. The accompanied peaks around 877 cm^−1^, 815 cm^−1^, and 750 cm^−1^ were associated with the bending vibration of C–H in the aromatic rings [26]. The molecular chain of hydrocarbons may be inferred from the stretching vibration of aliphatic C–H at 2914 cm^−1^ and 2865 cm^−1^ or the bending vibration of aliphatic –CH_2_ at 1431 cm^−1^ and –CH_3_ at 1386 cm^−1^ [27].

The peaks of uncured 20%-BPF resin related to the above functional groups were remarkable, as shown in Figure 3A. It is demonstrated that hydroxymethyl groups can be produced by the natural polyphenols of bio-oil, which was able to improve the crosslinking degree. Besides, adding bio-oil favored the formation of more hydrocarbon chains during the synthesis process, which could modify the brittleness. However, the peaks of uncured 40%-BPF resin mentioned above became weaker, which suggested that adding too much bio-oil would severely impact the inherent molecular structure of PF resin.

The FTIR spectrum of the cured BPF and PF resins is illustrated in Figure 3B. The band corresponding to phenolic hydroxyl and hydroxymethyl became weak, indicating that hydroxymethyl was consumed and a condensation reaction took place between phenolic hydroxyls during the curing process. The peaks associated with hydrocarbons decreased notably, suggesting that they may be consumed through the reaction with hydroxymethyl or phenolic hydroxyl [27]. The typical vibrations of benzene ring and aromatic ring disappeared, demonstrating that benzene rings got close to each other and a multi-benzene fused ring structure formed that weakened the vibration of the benzene skeleton.

Compared to cured PF resin, the intensity of the peaks related to aliphatic C–H, –CH_2_, and –CH_3_ were strong in cured 20%-BPF resin, proving that many hydrocarbons were present after the curing process. This demonstrated that adding bio-oil had toughening effects, and its initial mechanism was revealed. The above results are consistent with the performance of the oxygen index and bending strength of various BPF resins, as shown in Table 1. 

### 3.5. SEM Analysis 

**Figure 4:**
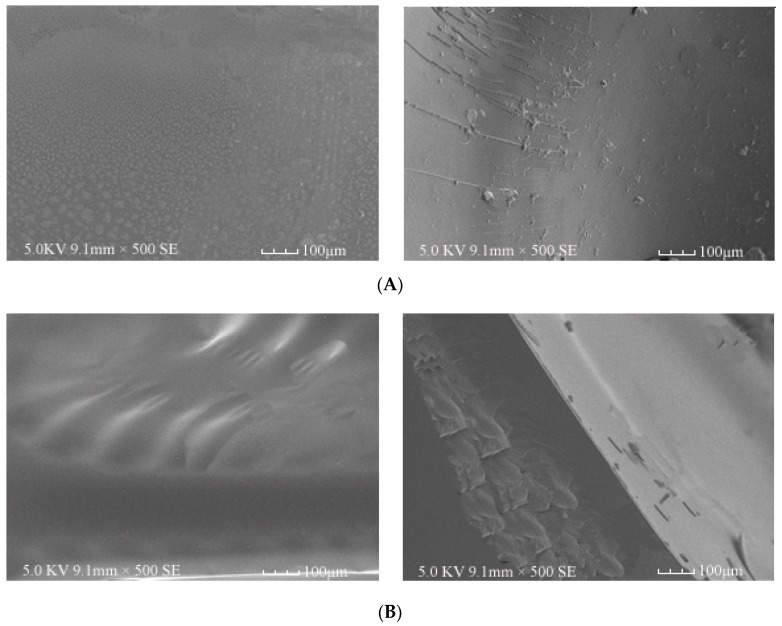

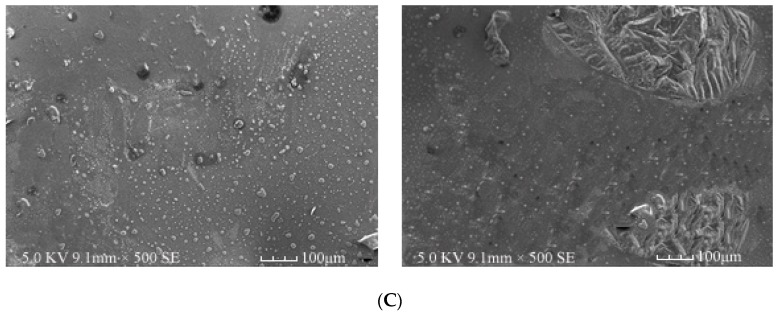
SEM images of (**A**) cured PF resin; (**B**) cured 20%-BPF resin; and (**C**) cured 40%-BPF resin.

The SEM images of the surface of cured PF resin and cured BPF resins with bio-oil addition of 20% and 40%, respectively, are shown in Figure 4A–C. Though the surface appeared to be uniform and smooth, the cured PF resin was featured by brittle failure because line shape cracks and solid particles were observed. It was not only smooth and flat; some corrugations were present in the surface of cured 20%-BPF resin, indicating that a stronger intermolecular force was formed in the crosslinking structure of the resin. The surface of 40%-BPF was rough and included many solid particles and tiny holes, which may be caused by carbide or other compounds in bio-oil which would not take part in the synthesis reaction.

## 4. Conclusions

That use of bio-oil from fast pyrolysis and paraformaldehyde as raw materials was an effective approach for synthesizing BPF resins—a desirable resin for fabricating phenolic-based material. The BPF resin with the bio-oil addition of 20% had good performance in oxygen index and bending strength, indicating that adding bio-oil was beneficial to modify the fire resistance and brittleness of PF resin. Adding bio-oil had an impact on curing characteristics and thermal degradation process of PF resin, but the influence was insignificant when the addition was relatively low. When the addition of bio-oil was 20%, more chains of hydrocarbons were formed in BPF resin. This showed that the crosslinking degree of cured 20%-BPF resin was improved and the intermolecular force of crosslinking structure was enforced.

## Figures and Tables

**Table 1:** Properties and resin casting model performances of bio-oil phenol formaldehyde (BPF) resin.

Resin	Resin Properties				Casting Model Performances			
	Viscosity (mPa·s)	Solid Content (%)	Free Formaldehyde (%)	Free Phenol (%)	Density (g·cm^−3^)	Oxygen Index (%)	Bending Strength (MPa)	MOE ^1^ (MPa)
PF	915	80.3	1.7	2.7	1.32	64.8	20.4	836.7
10%-BPF	862	78.9	1.2	3.2	1.31	89.2	29.7	1198.2
20%-BPF	728	76.8	1.1	3.4	1.30	93.1	41.2	1426.3
30%-BPF	586	74.3	0.9	3.9	1.30	88.6	25.6	1087.8
40%-BPF	462	71.9	0.8	4.3	1.29	82.5	22.8	986.3

^1^ Modulus of elasticity was designated as MOE.

**Table 2:** Thermal cure kinetics parameters of various BPF resins.

Resin	T_p_ (°C)				Curing Temperature (°C) ^1^	E_k_ (kJ mol^−1^) ^2^	E_f_ (kJ mol^−1^) ^3^
	5 °C·min^−1^	10 °C·min^−1^	15 °C·min^−1^	20 °C·min^−1^			
PF	118.1	128.5	136.5	144.8	110.0 (0.9962)	64.36 (0.9815)	67.59 (0.9850)
10%-BPF	127.7	137.2	147.9	153.3	119.7 (0.9838)	67.44 (0.9794)	70.66 (0.9831)
20%-BPF	130.2	139.5	148.7	155.2	122.4 (0.9936)	71.58 (0.9817)	74.64 (0.9849)
30%-BPF	137.8	146.9	153.3	160.1	131.2 (0.9933)	86.01 (0.9858)	88.45 (0.9880)
40%-BPF	140.5	148.5	155.7	161.3	134.1 (0.9940)	91.84 (0.9847)	94.03 (0.9869)

^1^ Curing temperature was calculated based on linear regression analysis of peak temperature at various heating rates; ^2^ E_k_ was calculated according to the Kissinger method; ^3^ E_f_ was calculated according to the Flynn–Wall–Ozawa method. The values in parentheses are the regression coefficients.
